# Should the WHO withdraw support for mass deworming?

**DOI:** 10.1371/journal.pntd.0005481

**Published:** 2017-06-08

**Authors:** Kevin Croke, Joan Hamory Hicks, Eric Hsu, Michael Kremer, Edward Miguel

**Affiliations:** 1 World Bank, Washington DC, United States of America; 2 Harvard T. H Chan School of Public Health, Boston, Massachusetts, United States of America; 3 Center for Effective Global Action, University of California, Berkeley, Berkeley, California, United States of America; 4 Department of Economics, University of California, Berkeley, Berkeley, California, United States of America; 5 Department of Economics, Harvard University, Cambridge, Massachusetts, United States of America; Australian National University, AUSTRALIA

In April 2016, the World Health Organization (WHO)’s Control of Neglected Tropical Diseases (NTD) department and Nutrition for Health and Development (NHD) department convened a Guidelines Development Group meeting to review the WHO’s recommendations for the control of soil-transmitted helminths in high-risk groups. Subsequent to this meeting, the WHO will announce whether it will reaffirm its long-standing recommendation of mass drug administration (MDA) in areas with more than 20% prevalence of soil-transmitted helminths (hookworm, whipworm, and roundworm). We recently released a new meta-analysis [1] working paper focusing on the effect of MDA on weight gain for children, which was presented at this WHO convening.

There is consensus that the relevant drugs are safe and effective. Indeed, they are the standard of care for those known to be infected. Since individual collection and testing of stool samples prior to treatment is prohibitively expensive and logistically impractical in many low-income contexts, the relevant question for policy is whether the expected benefits of MDA exceed its costs, taking into account uncertainty.

The literature on long-run educational and economic impacts of deworming of children in several high-prevalence settings suggests that this is the case [2]. However, the 2015 Cochrane review meta-analysis [3] disputes this view. They estimate that treatment of those known to be infected increases weight by 0.75 kg (95% confidence interval 0.24–1.26, *p* = 0.0038) but conclude that there is “substantial evidence” of no impact of mass treatment on weight or other outcomes at community level. This has led some to suggest that the long-term impacts discussed above are logically impossible and that the WHO policy is mistaken [4].

The new meta-analysis [1] working paper first examines statistical power in the recent Cochrane Review on deworming [3]. Next, we update the analysis in [3] by including studies omitted from that analysis and extracting additional data from included studies. To do this, we follow procedures outlined in the *Cochrane Handbook for Systematic Reviews of Interventions* [5], such as deriving standard errors from *p* values when the standard errors are not reported in the original article and contacting original study authors for clarification when necessary. The updated, full sample in [1] includes twice as many trials as analyzed by [3], substantially improving statistical power.

We find that the 2015 Cochrane Review analysis [3] is statistically underpowered; that is, its analysis of nutritional outcomes would likely conclude that MDA has no effect even if the true effect were large enough (given relevant treatment cost data) to be cost-effective relative to other interventions in similar populations. The hypothesis of a common zero effect of multiple-dose MDA deworming on child weight at longest follow-up is rejected at the 10% level using the dataset in [3], and with a *p* value <0.001 using the full sample in [1]. Adding any 1 of 5 individual updates to the data in [3] in isolation leads to rejection of the null hypothesis at the 5% level. Applying either of 2 study classification approaches used in previous Cochrane Reviews (prior to [3]) also leads to rejection at the 5% level.

In the full sample in [1], including studies in environments where prevalence is low enough that the WHO does not recommend deworming, the average effect on child weight is 0.134 kg ([95% CI: 0.031–0.236], random effects estimation). The random effects approach allows the true effect to vary from study to study due to contextual factors. Results are robust to removing any individual study. In environments with greater than 20% prevalence, where the WHO currently recommends mass treatment, the average effect on child weight is 0.148 kg ([CI: 0.039–0.258], random effects). In environments with more than 50% prevalence, where the WHO recommends multiple annual doses, the estimated effect is 0.182 kg ([CI: 0.070–0.293], random effects).

The implied average effect of MDA on infected children in the full sample in [1] (calculated by dividing estimated impact by worm prevalence for each study and applying a random effects meta-analysis model) is 0.301 kg. This likely reflects considerably larger effects on those with moderate- to severe-intensity infections and smaller effects on those with light infections. For context, the difference in weight gain over 1 year for boys at the 25th versus at the 50th percentile of the weight-for-age distribution between ages 3 and 4 is 0.2 kg [6]. Moreover, since light infections are often asymptomatic and only 2%–16% of the population experience moderate- to severe-intensity infections in the studies in our meta-analysis that report this information, implied effects in the subpopulation of those with moderate- or severe-intensity infections are likely much larger. There is thus consistency between the estimated range [3] report for the impact of treatment on those known to be infected and the ranges estimated in studies of MDA. At 0.22 kg per US dollar, the estimated average weight gain per dollar expenditure from deworming MDA (assuming 2 annual treatments) is more than 35 times that from school feeding programs, as estimated in randomized controlled trials (RCTs) [1].

While the WHO decision will be based on health benefits, we believe that it is also worth noting recent research that provides new evidence on the long-run educational and economic benefits of deworming. Baird et al. [7] estimate that a decade after treatment, males who participated in mass deworming in Kenya worked 17% more hours per week and had higher living standards, missing 1 fewer meals per week. Girls were approximately one-quarter more likely to have passed the primary school leaving exam and attended secondary school. The estimated value of benefits exceeds the cost by more than 100-fold. While the results of any 1 study should not be taken in isolation and effects may differ across environments, even if policy makers believed there was a 1 in 100 chance of experiencing effects of this magnitude, the expected benefits of deworming would exceed the costs. Ahuja et al. [2] summarize 3 other studies (2 working papers submitted for publication and 1 published) that also find substantial long-run educational and economic benefits [8, 9, 10]. The existence of positive child-weight effects of mass deworming estimated in [1] helps to provide a plausible nutritional channel for these long-run impacts.

In light of the mounting evidence on both the short-run impacts on child weight and long-run educational and economic effects of deworming, we believe that the expected benefits of deworming are likely to greatly exceed the cost, and that the long-standing support of WHO and other international donors and organizations for mass deworming remains scientifically justified.
